# Stromal interaction molecule 1 (STIM1) knock down attenuates invasion and proliferation and enhances the expression of thyroid-specific proteins in human follicular thyroid cancer cells

**DOI:** 10.1007/s00018-021-03880-0

**Published:** 2021-06-21

**Authors:** Muhammad Yasir Asghar, Taru Lassila, Ilkka Paatero, Van Dien Nguyen, Pauliina Kronqvist, Jixi Zhang, Anna Slita, Christoffer Löf, You Zhou, Jessica Rosenholm, Kid Törnquist

**Affiliations:** 1 Minerva Foundation Institute for Medical Research, Biomedicum Helsinki 2U, Tukholmankatu 8, 00290 Helsinki, Finland; 2 Faculty of Science and Engineering, Cell Biology, Åbo Akademi University, Tykistökatu 6A, 20520 Turku, Finland; 3 Turku Bioscience Centre, University of Turku and Åbo Akademi University, 20520 Turku, Finland; 4 Division of Infection and Immunity, School of Medicine, Systems Immunity University Research Institute, Cardiff University, Cardiff, UK; 5 Department of Pathology, University of Turku, Turku, Finland; 6 College of Bioengineering, Chongqing University, No. 174 Shizheng Road, Chongqing, 400044 China; 7 Pharmaceutical Sciences Laboratory, Faculty of Science and Engineering, Åbo Akademi University, BioCity, Artillerigatan 6A, 20520 Turku, Finland; 8 Research Centre for Cancer, Infections and Immunity, Institute of Biomedicine, University of Turku, Kiinamyllynkatu 10, 20520 Turku, Finland

**Keywords:** Thyroid cancer, Stromal interaction molecule 1 (STIM1), ORAI1 calcium channel, Store-operated calcium entry (SOCE), Migration, Invasion, Proliferation

## Abstract

Stromal interaction molecule 1 (STIM1) and the ORAI1 calcium channel mediate store-operated calcium entry (SOCE) and regulate a multitude of cellular functions. The identity and function of these proteins in thyroid cancer remain elusive. We show that STIM1 and ORAI1 expression is elevated in thyroid cancer cell lines, compared to primary thyroid cells. Knock-down of STIM1 or ORAI1 attenuated SOCE, reduced invasion, and the expression of promigratory sphingosine 1-phosphate and vascular endothelial growth factor-2 receptors in thyroid cancer ML-1 cells. Cell proliferation was attenuated in these knock-down cells due to increased G1 phase of the cell cycle and enhanced expression of cyclin-dependent kinase inhibitory proteins p21 and p27. STIM1 protein was upregulated in thyroid cancer tissue, compared to normal tissue. Downregulation of STIM1 restored expression of thyroid stimulating hormone receptor, thyroid specific proteins and increased iodine uptake. STIM1 knockdown ML-1 cells were more susceptible to chemotherapeutic drugs, and significantly reduced tumor growth in Zebrafish. Furthermore, STIM1-siRNA-loaded mesoporous polydopamine nanoparticles attenuated invasion and proliferation of ML-1 cells. Taken together, our data suggest that STIM1 is a potential diagnostic and therapeutic target for treatment of thyroid cancer.

**Supplementary Information:**

The online version contains supplementary material available at 10.1007/s00018-021-03880-0.

## Introduction

Thyroid cancer is the most prevalent endocrine cancer, the incidence and related mortality of which are increasing globally [1]. 95% of thyroid cancer forms arise from follicular cells [2, 3]. Usually, the prognosis of thyroid tumors is good. However, due to dedifferentiation or genetic mutations, highly aggressive phenotypes arise, which potentiate metastasis of tumor cells mainly to lungs, brain and bones, causing death of the patient within 6 months [4, 5]. There is no rational treatment available for such tumors [6]. Thus, novel approaches to curtail and halt the progression of thyroid cancer to a metastatic and terminally fatal disease are urgently warranted.

Store-operated calcium entry (SOCE) is activated as a result of agonist-evoked emptying of calcium stores in the endoplasmic reticulum (ER). This mechanism results in activation of plasma membrane calcium channels and calcium entry into the cells, ensuring that the calcium stores will be replenished [7]. The calcium signals generated by the SOCE regulate a multitude of cellular functions, including cancer cell proliferation, invasion and migration [8–11].

Stromal interaction molecule 1 (STIM1) is localized in the ER and plasma membrane. It functions as an ER-calcium sensor protein and gets activated by ER calcium-depletion, oligomerizes with other STIM1 molecules, and recruits plasma membrane ORAI1 channels. This results in calcium entry into the cells and refilling of the ER-calcium stores [12]. STIM1 has been shown to activate also other channels, e.g. transient receptor potential canonical (TRPC) isoforms, including TRPC1 [13]. Previously, we have reported that TRPC1 functions in thyroid cancer cells as a receptor-operated calcium channel and modulates thyroid cancer cell invasion [14].

Previous studies have indicated that there are relatively high expression levels of STIM and ORAI isoforms in several cancer cell types [15]. The expression and function of STIM1 and ORAI1 in thyroid cancer progression and invasion have, however, remained elusive.

In the present study, we investigated the expression of STIM1 and ORAI1 proteins in thyroid cells and their importance in regulating invasion. We report that the expression of STIM1 is upregulated in all thyroid cancer cell lines studied compared to primary thyroid cells. Notably, STIM1 was upregulated in all thyroid cancer patient tissue samples investigated, as compared with normal thyroid tissue. In thyroid follicular ML-1 cancer cells, SOCE was significantly decreased in both stable STIM1 (STIM1-KD) and ORAI1 (ORAI1-KD) knock-down cells, compared with control mock-transfected cells. Furthermore, the basal invasion was decreased, and the sphingosine 1-phosphate (S1P)-evoked invasion was abolished in ML-1 cells after STIM1-KD or ORAI1-KD. In STIM1-KD cells, proliferation was reduced and the expression of S1P_3_, vascular endothelial growth factor-2 (VEGFR2), and H1F-1α was decreased, as well as the activity of matrix metalloproteinase -2 and -9 (MMP2 and MMP9). In a zebrafish xenograft model, STIM1-KD decreased tumor growth. Furthermore, we show that STIM1-KD increases the susceptibility of thyroid cancer ML-1 cells to drugs used for treatment of thyroid cancer, reinstates the expression of thyroid stimulating hormone receptor (TSHR), thyroid specific proteins and increased iodine uptake. Finally, use of modified mesoporous polydopamine (MPDA) nanoparticles was found to be a useful system for functional delivery of siRNA in thyroid cancer cells.

## Materials and methods

H6621 cell culture medium, supplements kit and gelatin-based coating solution were purchased from Cell Biologics (Chicago, IL, USA). DMEM, sodium pyruvate solution, BSA, fatty acid-free BSA, poly-l-lysine, puromycin, HEPES solution and JumpStart *Taq *DNA polymerase were purchased from Sigma-Aldrich (St. Louis, MO, USA). RPMI 1640 medium (without l-glutamine) was from Lonza (Basel, Switzerland). FBS, trypsin–EDTA solution, l-glutamine, penicillin/streptomycin (P/S), OptiMEM, and F-12 (Ham’s nutrient medium) GIBCO™, RevertAid reverse transcriptase, RiboLock RNase inhibitor, random hexamer primers, dNTPs, GeneRuler 100-bp Plus DNA Ladder, HEPES, the bicinchoninic acid protein assay reagent kit Pierce™ and Alexa Fluor™ Red Fluorescent Control siRNA were from Thermo Fisher Scientific (Waltham, MA, USA). Sphingosine 1-phosphate (S1P) was from Biomol (Plymouth, PA). Primary antibodies against S1P_1_, S1P_3_, TG, NIS, TPO, and Hsc70 were from Santa Cruz Biotechnology, Inc. (Santa Cruz, CA). HRP-conjugated goat anti-rabbit IgG and the Aurum total RNA isolation kit were from Bio-Rad (Hercules, CA, USA). Primary antibodies against VEGFR2, HIF-1α, p21waf1/cip1, p27kip1, cdk6, MMP2, ERK1/2, pERK1/2, E-cad, N-Cad, TSHR, and HRP-conjugated anti-rat and anti-mouse IgG were from Cell Signaling Technology (Denver, MA, USA). Primary antibody against STIM1, ORAI1, and calpain activity and MTT proliferation assay kits were purchased from Abcam (Cambridge, MA). Cell culture plastic ware and human collagen type IV were from Becton Dickinson (Franklin Lakes, NJ, USA), and Transwell inserts for invasion assays were from Corning, Inc. (Corning, NY). All the chemicals and reagents used were of molecular biology and reagent grades. Fura-2 AM was from Molecular Probes (Eugene, OR). Thapsigargin was from Alexis Corporation (San Diego, CA). KAPA Probe Fast Master Mix was from Kapa Biosystems (Boston, MA), and the Universal Probe Library probes were from Roche (Basel Switzerland).

### Cell culture

Human primary thyroid epithelial cells were purchased from Cell Biologics (Chicago, IL, USA). The cells were cultured in complete human epithelial cell medium with supplements kit (H6621, cell biologics, Chicago, IL, USA). The cells were plated on gelatin-coated plates as per manufacturer’s instructions. The human ML-1 follicular thyroid cancer cells were cultured in DMEM with 10% FBS, 1% penicillin/streptomycin (P/S), and 1% L-glutamine. FTC-133 thyroid follicular cancer cells were purchased from Banca Biologica e Cell Factory (Genova, Italy). The cells were cultured in DMEM and F-12 (Ham’s) medium (1:1) with 10% FBS and 1% L-glutamine and 1% P/S. The C643 anaplastic thyroid cancer cells were provided by Dr Nils-Erik Heldin (Karolinska Institute, Stockholm, Sweden) and cultured in DMEM with 10% FBS, 1% L-glutamine, and 1% penicillin/streptomycin. The anaplastic thyroid cancer THJ-16T cells were acquired from Dr John Copland (Mayo Clinic, FL, USA). The cells were cultured in RPMI 1640 with 10% FBS, 1% penicillin/streptomycin, 1 mM sodium pyruvate, and 25.03 mM HEPES. The cell cultures were maintained in a water saturated atmosphere supplemented by 5% CO2 and 95% air at 37 °C in the incubators.

### Generation of stable cell lines

Cells were grown on 12-well plates. The transduction was performed according to the manufacturer’s instructions using non-targeting shRNA lentivirus particles, and STIM1- or ORAI1-targeting lentiviral particles (Sigma, St. Louis, MO, USA). The sequences are provided in Supplementary Table 1. After 48 h, the medium was changed to medium containing 0.5 µg/ml Puromycin. The cells were cultured with puromycin-containing medium hereafter. The knock-down of STIM1 and ORAI1 was measured on mRNA level by quantitative-PCR and on protein level by western blotting, respectively.

### Transient transfections

4 million cells were pelleted and re-suspended in 400 µl OptiMEM together with 20 µg of the control siRNA, siSTIM1, pYFP, pSTIM1 or pORAI1 plasmids. The cells were electroporated at 975 µF and 240 V and were grown in respective media for 48 h before the start of the experiments.

### Qualitative end-point PCR and quantitative realtime PCR

RNA was extracted with Aurum™ Total RNA Mini Kit (Bio-Rad; CA, USA) or by TRI reagent (Sigma Aldrich; St. Louis, MO, USA) according to the manufacturer’s instructions. RNA integrity was checked by gel electrophoresis and RNA concentration and purity was determined with Nanodrop 2000 (Thermo Fisher Scientific; Waltham, MA) and NanoVue Plus (Healthcare Bio-Sciences AB; Uppsala, SE). cDNA samples were prepared with RevertAid reverse transcriptase and SuperScript IV^®^ Reverse Transcriptase (Thermo Fisher Scientific, MA, USA) from equal amounts of RNA. Reaction mixtures lacking either reverse transcriptase or RNA were used as negative controls. For primers information see supplementary Table 1. Quantitative real-time PCR assays were designed with the Universal Probe Library (UPL) Assay Design Center (www.rocheappliedscience.com). PBGD was used as a reference gene. RT-PCR was performed with KAPA Probe Fast Master Mix and the StepOnePlus Real-Time PCR System (Applied Biosystems™, Thermo fisher scientific, MA, USA) using the relative standard curve method.

### Western blotting

The whole cell lysates and for the Western blotting were performed as described elsewhere [16]. In some experiments, equal amounts of proteins were loaded on 10% Fast Cast Stain-free sodium dodecyl sulfate polyacrylamide gel electrophoresis (SDS-PAGE) gels. Blotting was performed using PVDF membranes using a BioRad Transblot system. After blocking, the blots were probed overnight with primary antibodies. Proteins were detected with enhanced chemiluminescence (ECL; Thermo Scientific, 242 Waltham, MA). Densitometric analysis was performed using the ImageJ program for image analysis (National Institutes of Health, Bethesda, MD), and the results were corrected for protein loading by normalization with Hsc70 expression.

### Measurements of [Ca^2+^]_i_ in single cells

Cells were processed and analyzed as described elsewhere [17]. A HAMAMATSU digital camera C10600 ORCA-*R*^2^ with controller (Photonics K.K.) was used to capture fluorescence images at 1–3 s to avoid bleaching. The images were acquired and processed using the Axon Imaging Workbench 6 software (INDEC BioSystems, Santa Clara, CA). The intracellular free calcium concentrations were calculated as the ratio F_340_/_380_.

### Invasion assays

The invasion assays were performed as described elsewhere [18]. Cells were serum starved by growing in 0.2% FAF-BSA-containing serum-free medium (SFM) overnight before the start of the invasion assays. In these experiments, the cells were stimulated with S1P and allowed to migrate towards 10% lipid-stripped FBS (LS-FBS) for 16 h. In other experiments, cells were allowed to invade towards 10% FBS-containing culture medium for 16 h. The non-migrated cells were removed with a cotton swab. The migrated cells were fixed in 2% paraformaldehyde for 10 min and then stained with 0.1% crystal violet in 20% methanol for 5 min. The membranes were washed with PBS and water and allowed to dry overnight. The cells were counted at 40X magnification in a straight line bisecting the membrane.

### Zymography

ML-1 MOCK and STIM1-KD cells were grown on 35-mm plates up to 80% confluency. The medium was changed to SFM medium for overnight incubation. Next day, the medium was changed to fresh SFM medium (1 ml on each plate) and the cells were stimulated with or without S1P (100 nM) for 6 h. After 6 h, the media were collected. Equal volumes of medium were mixed with loading buffer (0.1 m Tris–phosphate buffer, pH 6.8, containing 20% glycerol, 6% SDS, and 0.04% bromphenol blue). The samples were electrophoresed with the 10% SDS gels containing gelatin (2.65 mg/ml). The gels were incubated in Zymo buffer (50 mm Tris–HCl containing 2.5% Tween 80 and 0.02 NaN_3_, pH 7.5) for 30 min. The gels were then incubated in Zymo buffer containing 1 μm ZnCl_2_ and 5 mm CaCl_2_ for 30 min. For gelatinolytic activity gels were incubated at 37 °C for overnight in buffer containing 50 mm Tris–HCl, 5 mM CaCl_2_, 1 μm ZnCl_2_, and 0.02% NaN_3_ (pH 7.5). The gelatin degradation was visualized under UV light, and after that, the gels were stained with Coomassie Blue R250 for 1–2 h. The gelatinolytic activity was visualized as clear bands against blue background on stained gels. The clear bands, after destaining for 30 min as shown in (Fig. 5E), were quantified by the program ImageJ. The data were normalized with the respective total protein concentrations of the respective cells in the culture plates.

### Calpain activity assays

2 million cells were grown on 100-mm plates. The cells were serum-starved overnight and stimulated with or without S1P (100 nM) for 6 h. Next, the cells were detached and washed three times with PBS. Thereafter, calpain activity assays were performed according to the manufacturer’s instructions (Abcam, Cambridge, MA). The samples were analyzed at an excitation of 400 nm and emission at 505 nm using a fluorescence analyzer. The results were normalized with the respective total protein concentrations of each culture plate and presented as % calpain activity.

### Proliferation assays

The *[*^*3*^*H] thymidine incorporation method* was used to study the proliferation of the ML-1 MOCK, STIM1-KD and ORAI1-KD cells. 50,000 cells were seeded on 35-mm plates and allowed to grow for 24 h. Four hours prior to the end of each experiment, 0.4 μCi/ml [^3^H]thymidine was added. The cells were washed three times with PBS, incubated for 10 min with 5% trichloric acetic acid, and then incubated for 10 min with 0.1 m NaOH. The samples were transferred into scintillation tubes and high sample load scintillation cocktail Optiphase Hisafe 3 was added. The radioactivity was measured using a Wallac 1414 liquid scintillation counter.

#### MTT proliferation assay method 10,000

ML-1 MOCK and STIM1-KD cells were grown on 96 well plate for overnight and treated with indicated concentration of respective drugs for 24 h. Thereafter, the assays were performed according to the manufacturer’s instructions. The absorbances were measured at 590 nm using a spectrophotometer.

### FACS analysis

0.5 million ML-1 Mock, STIM1-KD and ORAI1-KD cells were grown overnight on 35-mm plates. The cells were detached with EDTA-trypsin solution and were centrifuged. The cell pellets were suspended in 500 μl of propidium iodide solution (0.05 mg/ml propidium iodide, 3.8 μM sodium citrate, 0.1% Triton X-100 in PBS) and incubated for 15 min at room temperature. The samples were then processed by flow cytometry using FACSCalibur and CellQuest Pro software (BD Biosciences, San Jose, CA). ModFit LT 4.1 software was used to calculate the percentage of cells in each phase of cell cycle.

### Iodine uptake assays

The uptake of iodide in ML-1 MOCK and STIM1-KD cells was determined by a nonradioactive, fast and highly reproducible spectrophotometric method based on the catalytic effect of iodide on reduction of yellow cerium (IV) to colorless cerium (III) in the presence of arsenious acid (Sandell-kolthoff reaction). The assay was performed exactly as described elsewhere [19]. In some experiments, the cells were pre-incubated with 1 mU/ml TSH for overnight before the start of the assay.

### Synthesis and modification of MPDA

The mesoporous polydopamine (MPDA) particles were prepared by a templated synthesis method as described elsewhere [20]. The surface of the obtained MPDA nanoparticles were modified by *N*,*N*-dimethylethylenediamine (DMEA) presenting a tertiary amine group for facilitating the siRNA loading [21]. 1 mg of MPDA nanoparticles were suspended in 2 ml of Tris buffer (pH.8.5). After the introduction of DMEA (25.6 mg), the mixture was stirred for 1 h at room temperature. The DMEA modified MPDA nanoparticles were isolated from the suspension by centrifugation.

### siRNA loading of DMEA modified MPDA nanoparticles

0.1 mg siRNA STIM1, non-target control siRNA or control siRNA-Alexa Fluro 555 were mixed with 1 mg DMEA modified MPDA in 1 ml of 2-(N-morpholino) ethanesulfonic acid (MES) buffer (final concentration 10 mM, pH 5.0) through continuous vibration mixing for 40 min. The siRNA loaded DMEA modified MPDA nanoparticles were separated by centrifugation at 11 000 rpm for 15 min and stored at − 20 °C. 50,000 ML-1 cells were grown on polylysine coated glass bottom 35 mm plate for overnight. Next day, 5 µM control siRNA loaded or siRNA STIM1 loaded nanoparticles were added to respective culture plates. The cells were grown for 96 h to 120 h. The cells were washed, and the coverslips were mounted with DAPI containing mounting media ProLong™ Gold Antifade mountant with DAPI. The laser scanning confocal microscopy was performed using a Leica TCS SP8 X microscope (Leica Microsystems GmbH, Wetzlar, Germany) with 63 × oil immersion magnification and a HC PL APO CS2 objective.

### Zebrafish embryo xenograft

The procedure to carry-out xenotransplantation in zebrafish embryos was modified from as described elsewhere [22, 23]. Adult zebrafish (Danio rerio, casper strain (*roy−/−; mitfa−/−*) [24] were maintained according to standard procedures [25]. The embryos were collected after natural spawning in breeding tanks. Experimentation with zebrafish was performed under license ESAVI/9339/04.10. 07/2016. The zebrafish embryos were cultured in E3-medium (5 mM NaCl, 0.17 mM KCl, 0.33 mM CaCl_2_, 0.33 mM MgSO_4_) supplemented with 0.2 mM phenylthiourea (PTU, Sigma-Aldrich) at 33 °C. Two days post-fertilization, the embryos were anesthetized with MS-222 (200 mg/l, Sigma-Aldrich) and mounted into low-melting point agarose for tumor transplantation.

Prior to transplantation, ML-1 cells were labelled with 5 µM CellTracker Green CMFDA (Thermofisher Scientific), trypsinized and washed with PBS. Approximately 5–10 nl of ML-1 cell suspension was microinjected into yolk of the embryo using CellTramVario (Eppendorf), Injectman Ni2 (Eppendorf) micromanipulator and self-made borosilicate glass needles pulled from glass capillaries (TW100-4, World Precision Instruments Ltd., Sarasota, FL, USA) using a micropipette puller (PB-7, Narishige, Tokyo, Japan). After injections, the agarose gel was broken and embryos were released, washed with E3 and cultured in E3-PTU at 33 °C. On 1 day post-injection (1 dpi), the successfully xenografted healthy embryos were selected to the experiment and placed into 12-well or 96-well plates (1 embryo per well). The embryos were anesthesized and imaged at 1 dpi and 4 dpi using a Zeiss AxioZoom fluorescence stereomicroscope of Nikon Eclipse widefield microscope.

The size of the tumor was determined by manually measuring the fluorescent tumor area using FIJI software (ImageJ version 1.49 k) [26]. Samples having significant malformations, died embryos and images where embryo was not laterally oriented or out-of-focus were excluded from the analysis. Samples were not blinded.

### Whole-mount immunofluorescence staining of zebrafish embryos

Embryos were fixed for 2 h at room temperature with 4% PFA in PBSTw (PBS + 0.2% Tween-20). The embryos were rinsed twice with PBSTw after fixation and premeabilized for 3 h with 2 percent Triton X-100 in PBS. After permeabilization, the embryos were blocked in blocking solution (PBSTw + 5% fetal calf serum) for overnight at + 4C. After blocking, the embryos were incubated with primary antibody (anti-Cleaved Caspase-3, #9664S, Cell Signaling Technology or anti-ki67, ab15580, Abcam) in blocking solution over night at + 4C. This was followed by 4 × 30 min washes with PBSTw and by subsequent incubation in secondary antibody (Alexa 568 donkey anti-rabbit, Invitrogen) in blocking solution for overnight at + 4C. Then samples were washed for 3 h at room temperature with PBSTw and mounted in 100% glycerol for imaging. The sample tubes were gently rotated throughout the staining process.

Images were captured using Leica SP5 matrix confocal microscope with 20x/0.7 air objective, 2 × zooming, 600 Hz scanning speed and 515 × 512 pixel image settings. Line averaging (value 3) and frame accumulation (value 3) were used while acquiring images. The acquired images were processed using FIJI software. Fluorescence images were subjected to filtering with Gaussian blur filter (radius 1) and to background removal (rolling ball radius 50). Finally, the brightness and contrast were linearly adjusted, and all compared samples were processed with similar settings. Tumor area was outlined with segmented line tool, and mean intensity on Cleaved Caspase 3 or ki67 channel was measured. Statistical analysis was carried out using Graph Pad Prism 6.05 software and nonparametric Mann–Whitney U-test. These experiments with whole-mount zebrafish xenografts were performed under license ESAVI/31414/2020.

### STIM1 staining of patient samples

The use of tissue samples and related data was approved by Auria Biobank (decision AB19-2988) and the Hospital District of Southwest Finland and Turku University Hospital (decision T12/011/20), and accordingly informed consent was obtained from all human participants. The samples were from both female and male patients (9 anaplastic thyroid carcinoma, 29 follicular thyroid carcinoma, and 138 papillary thyroid carcinoma). The age distribution of the patients was 18–88 years of age. Thyroid tissue was prepared according to standard histology practice, using buffered formalin (pH 7.0) for fixation and embedding tissue into paraffin blocks. Tissue microarrays (TMAs) were prepared from tumour area of each patient. As controls, normal thyroid tissue samples (control) were obtained from 104 patients. Tissue sections were cut and stained with anti-STIM1 antibody (1/200; Abcam 57,834) using Labvision Autostainer (Thermo-Fisher Scientific, Fremont, CA, USA) and detected with an Orion 2 step detection system using goat anti mouse/rabbit HRP + WellMed T100 HRP. Final images were acquired at 20X magnification using the case viewer software (HISTECH Ltd, Hungary).

### High throughput data mining and Gene expression analyses

The clinical and transcriptomic data of the Thyroid Carcinoma (THCA) cohort were downloaded from the GDC database (https://portal.gdc.cancer.gov/projects/TCGA-THCA). We included 502 thyroid tumors and 58 adjacent normal samples for transcriptome analysis. Among those tumor samples, 353 were papillary thyroid tumors while 106 were follicular thyroid tumors. To assess gene expression levels in normal thyroid samples, we retrieved gene expression data (as raw count values) of 563 thyroid samples from the Genotype-Tissue Expression (GTEx) (https://gtexportal.org/home) for comparative analysis of gene expression. Only genes that express in at least 50% of the samples were assessed. DESeq2 was used for differential expression analysis between thyroid cancer groups and their matched para-cancerous samples [27]. A Benjamini–Hochberg adjusted *p* value of < 0.05 was considered as statistical significance. The expression profiles of genes of interest (STIM1 and ORAI1) were also extracted from both retrieved datasets after performing variance stabilizing (VST) transformation for comparative analyses between cancerous tissues, cancer adjacent normal tissues, and controls**.**

### Statistical analysis

The results are presented as means ± the standard error of the mean from at least three independent measurements. Student’s *t* test was used when two means were compared. One-way analysis of variance and Bonferroni’s post hoc tests were applied when three or more means were compared. Kruskal–Wallis test, Kolmogorov–Smirnov test and Fisher exact test were used to analyze non-normally distributed data. GraphPad Prism 8 program (GraphPad Software Inc., San Diego, CA) was used for the statistical analyses. *p* values < 0.05 were considered statistically significant.

## Results

### The expression of STIM1 and ORAI1 channels is increased in thyroid cancer cell lines

**Fig. 1:**
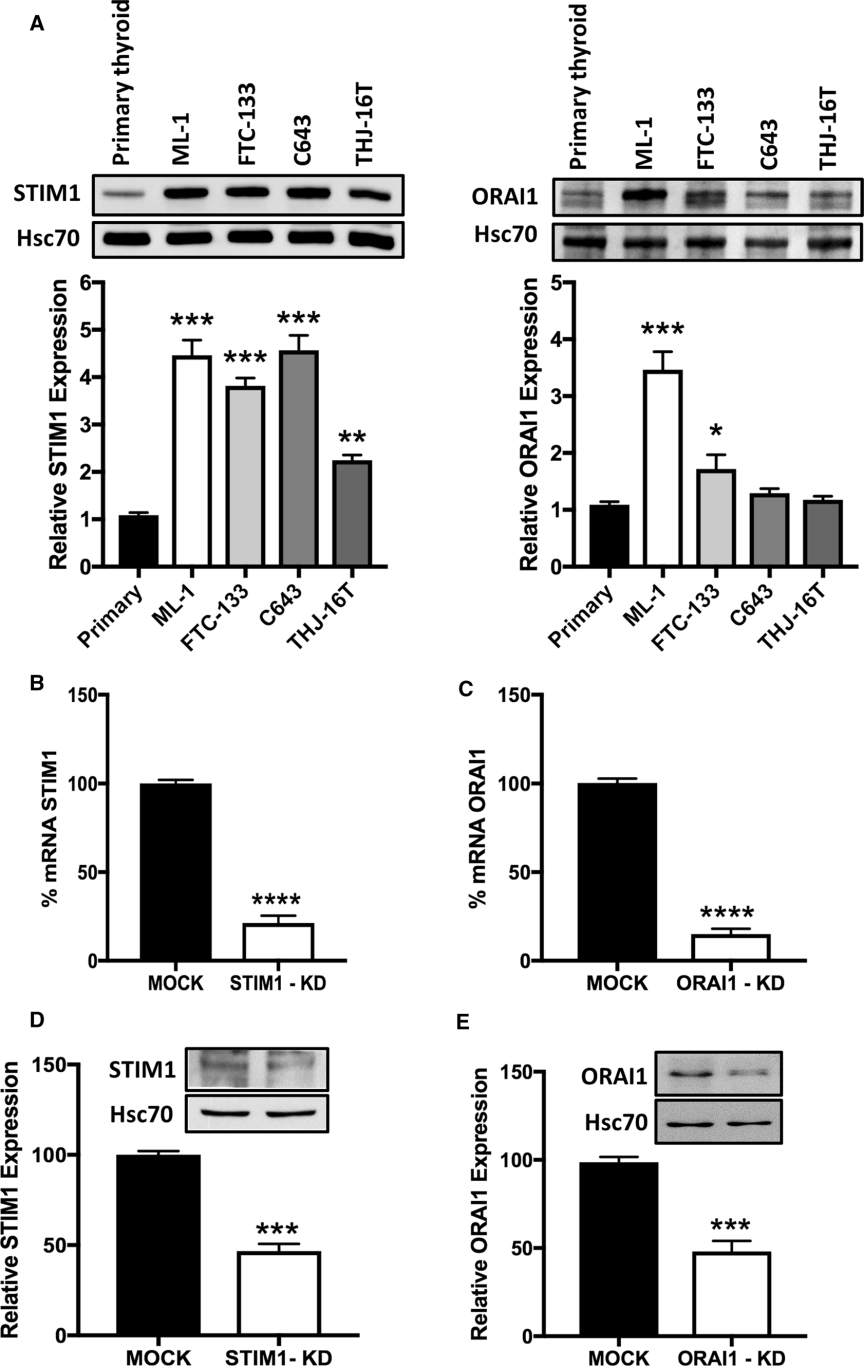
Expression of STIM1 and ORAI1 proteins and generation of stable knock-down cells. **A** Expression profile of STIM1 and ORAI1 proteins in primary thyroid and aggressive thyroid cancer cell lines by western blotting. The bars show the mean ± SEM (*n* = 3, *P < 0.05, **P < 0.01; ***P < 0.001). B-E, expression of STIM1 or ORAI1 in shRNA expressing (MOCK) and shRNA STIM1 expressing (STIM1-KD) or shRNA ORAI1 expressing (ORAI1-KD) cells on mRNA and protein levels by RT-PCR and Western blotting, respectively. The *bars *show the mean ± S.E. (*n* = 3). ***p < 0.001

Increasing evidence suggest that store-operated calcium entry (SOCE), mediated by the STIM1 and ORAI1 proteins, is a key player in regulating the pathophysiology of cancer [28, 29]. However, the expression and function of these key signaling proteins in human thyroid cancer has remained elusive. We show here that the protein level of STIM1 is higher in human follicular and anaplastic cancer cell lines, and that ORAI1 is higher in follicular cancer cell lines, compared with normal human thyroid primary cells (Fig. 1A). Based on these results, we then investigated the importance of the STIM1 and ORAI1 channels in follicular thyroid cancer ML-1 cells, a cell line showing the highest expression of STIM1 and ORAI1 compared to normal human thyroid cells. We generated stable STIM1 and ORAI1 knock-down (STIM1-KD, ORAI1-KD) ML-1 cell lines (Fig. 1B–E).

### Knock-down of STIM1 or ORAI1 attenuates store-operated calcium entry (SOCE)

**Fig. 2:**
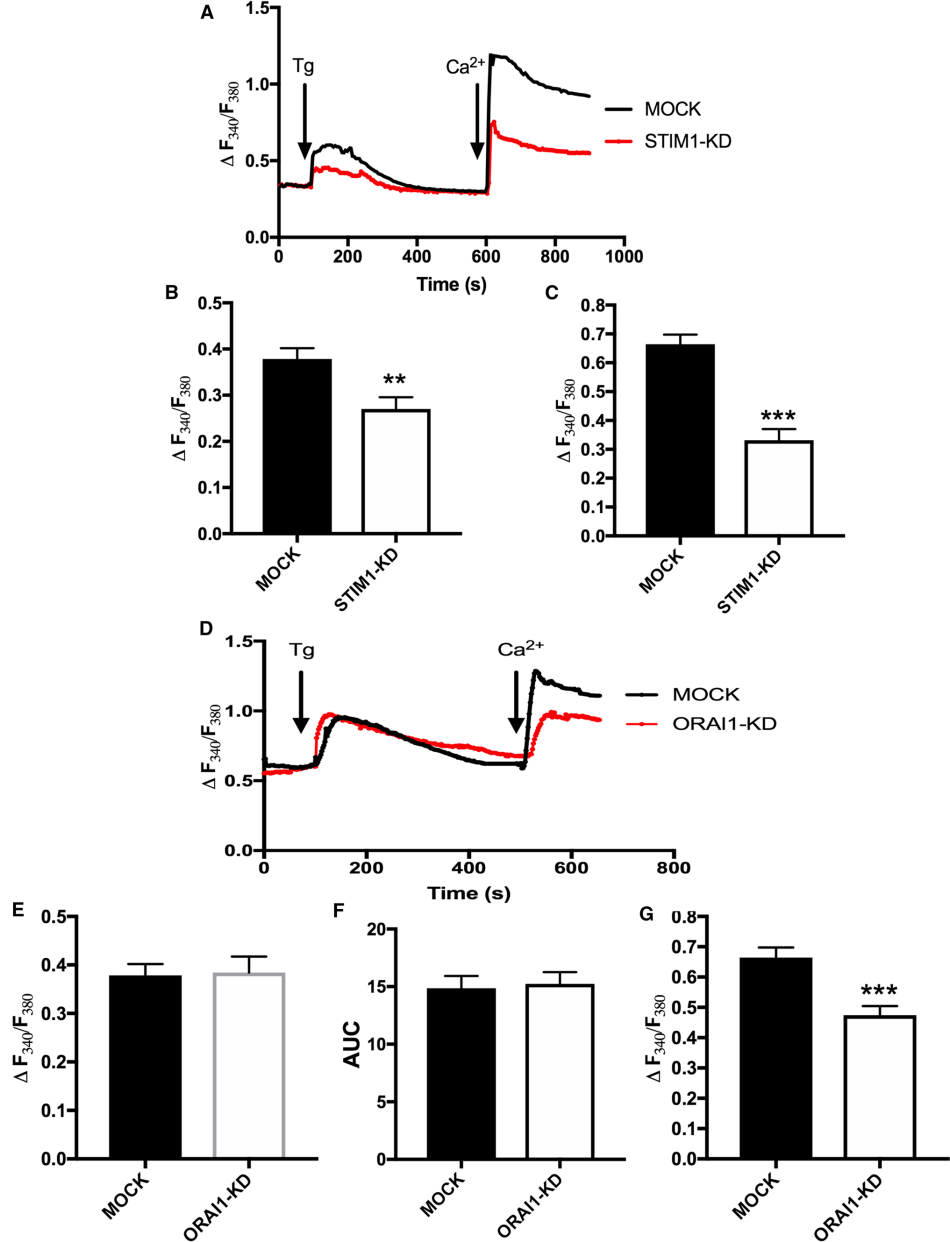
**A** Representative traces showing Thapsigargin-evoked (Tg, final concentration, 1 μM) changes in [Ca^2+^]_i_ in MOCK and STIM1-KD cells in calcium-free buffer and the effect of re-addition of 1 mM calcium (final concentration).**B** Bar diagram showing the magnitude of the Tg-evoked peak in [Ca^2+^]_i_. The bars show the mean ± S.E. of at least 40 cells. *p* < 0.01. **C** Bar diagram showing the magnitude of the change in [Ca^2+^]_i_ after re-addition of calcium (final concentration, 1 mM) to Tg-treated cells. The bars show the mean ± S.E. of at least 40 cells. ***p < 0.001. **D** Representative traces showing Tg-evoked (final concentration, 1 μM) changes in [Ca^2+^]_i_ in MOCK and ORAI1-KD cells in calcium-free buffer and the effect of readdition of 1 mM calcium. **E** Bar diagram showing the magnitude of the Tg-evoked peak in [Ca^2+^]_i_. The bars show the mean ± S.E. of at least 40 cells. **F** Bar diagram showing the area under the Tg curve (time, 60–480 s). The bars show the mean ± S.E. of at least 40 cells. **G** Bar diagram showing the magnitude of the change in [Ca^2+^]_i_ after readdition of calcium (final concentration, 1 mM) to Tg-treated cells. The bars show the mean ± S.E. of at least 40 cells. ***p < 0.001

Previous studies have established that STIM1 forms a complex with ORAI1 channels to evoke SOCE [8, 11]. Thus, we first investigated this mechanism in ML-1 cells. As can be seen in (Fig. 2A–C), a significant difference was observed in both the thapsigargin (Tg)-evoked calcium transient and on Ca^2+^ entry when Ca^2+^ was re-added to Tg-treated STIM1-KD cells, compared to the control MOCK cells. There was no significant difference in the Tg-evoked calcium transient in ORAI1-KD cells compared to MOCK cells as confirmed by calculating the area under the Tg-evoked calcium curve, as shown in (Fig. 2D–F). However, there was a significant decrease in Ca^2+^ entry when Ca^2+^ was re-added to the Tg-treated ORAI1-KD cells, compared to the MOCK cells (Fig. 2D, G). Thus, both STIM1 and ORAI1 are important in regulating SOCE in thyroid cancer cells.

### Knock-down of STIM1 or ORAI1 decrease invasion and receptor expression in thyroid cancer ML-1 cells

**Fig. 3:**
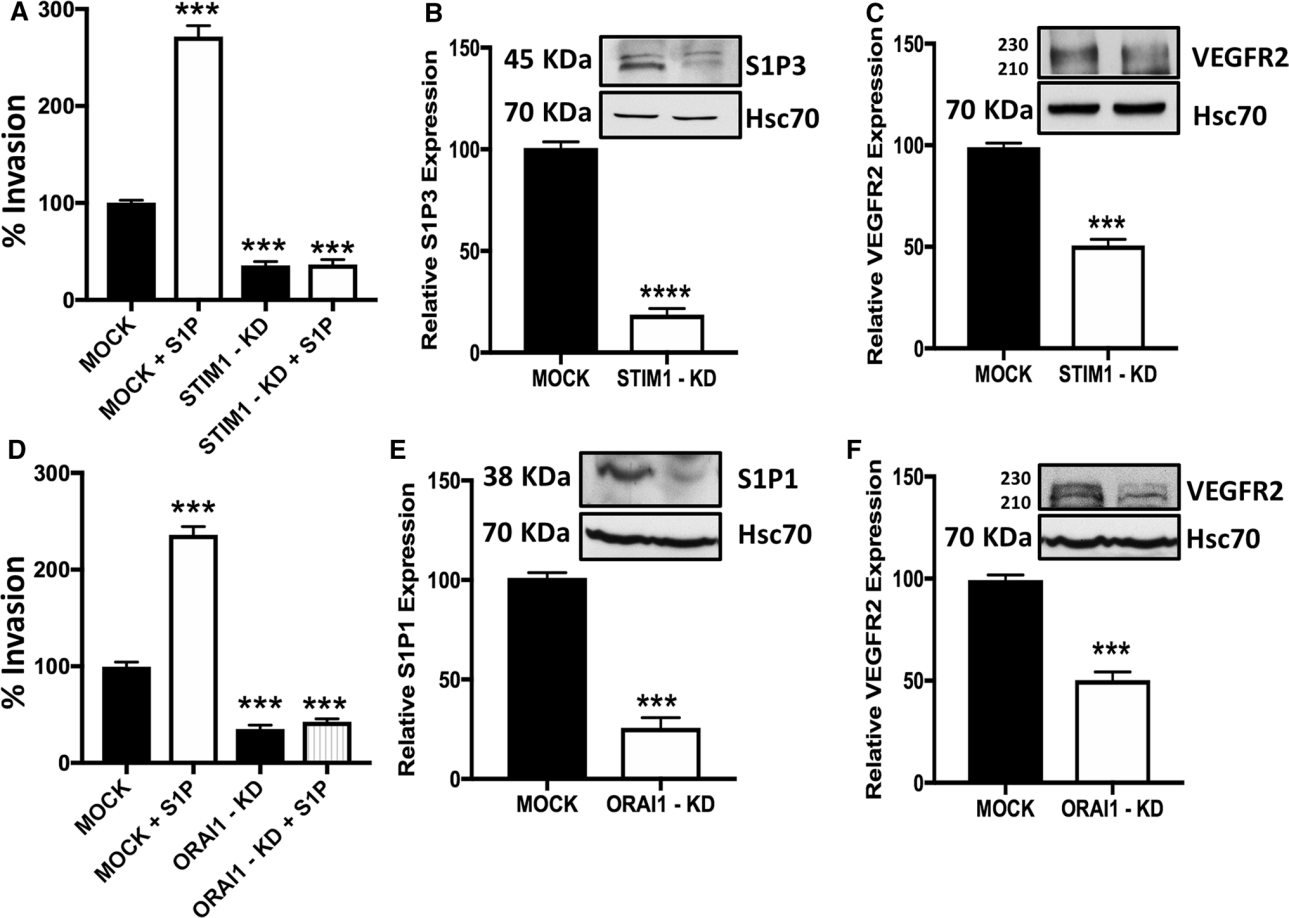
STIM1 or ORAI1 knock-down attenuates invasion and receptor expression in thyroid cancer ML-1 cells. **A**, **D**, STIM1 or ORAI1 knock-down both decreased the basal and abolished the S1P-evoked (final concentration, 100 nm) invasion in ML-1 cells. The *bars *show the mean ± S.E. (*n* = 4). *Denotes comparison with MOCK cells. ***p < 0.001. **B** STIM1 knock-down decreased the relative expression of S1P_3_. A representative Western blot is shown. The bar diagram shows the decreased expression of S1P_3_ in STIM1-KD cells compared with MOCK cells (mean ± S.E., *n* = 3) ***p < 0.001. **C** STIM1 knock-down decreased the relative expression of VEGFR2. A representative Western blot is shown. The bar diagram shows the decreased expression of VEGFR2 in STIM1-KD cells compared with MOCK cells (mean ± S.E., *n* = 3). ***p < 0.001. **E** ORAI1 knock-down decreased the relative expression of S1P_1_. A representative Western blot is shown. The bar diagram shows the decreased expression of S1P_1_ in ORAI1-KD cells compared with MOCK cells (mean ± S.E., *n* = 3). ***p < 0.001. **F** ORAI1 knock-down decreased the relative expression of VEGFR2. A representative Western blot is shown. The bar diagram shows the decreased expression of VEGFR2 in STIM1-KD cells compared with MOCK cells (mean ± S.E., *n* = 3). ***p < 0.001

STIM1 and ORAI1 have been reported to regulate invasion of cancer cells [29, 30]. We have previously characterized sphingosine 1-phosphate (S1P) and VEGFR2 signaling in thyroid cancer cells. S1P activates invasion of ML-1 cancer cells via the promigratory receptors S1P1, S1P3 and VEGFR2 [16, 31]. Thus, we investigated the effects of STIM1 or ORAI1 knock-down on ML-1 cancer cell invasion. As can be seen in (Fig. 3A, D), both basal and S1P-evoked invasion was attenuated in both the STIM1-KD and ORAI1-KD cells, compared with MOCK cells. Interestingly, the re-expression of STIM1 in STIM1-KD cells significantly restored the basal and S1P-evoked invasion, compared to MOCK cells. Likewise, re-expressing ORAI1 in ORAI1-KD cells significantly restored the basal and S1P-evoked invasion (supplementary Fig. 1A and B). To further explain this abolished effect of S1P on STIM1-KD and ORAI1-KD cells, we determined S1P and VEGFR2 receptors expression. The results showed that S1P3 and VEGFR2 were downregulated in STIM1-KD cells (Fig. 3B, C). In ORAI1-KD cells, S1P1 and VEGFR2 were downregulated (Fig. 3E, F). Thus, both STIM1 and ORAI1 are important regulators of thyroid cancer cell invasion.

### Knock-down of STIM1 and ORAI1 modulate ML-1 cell proliferation

**Fig. 4:**
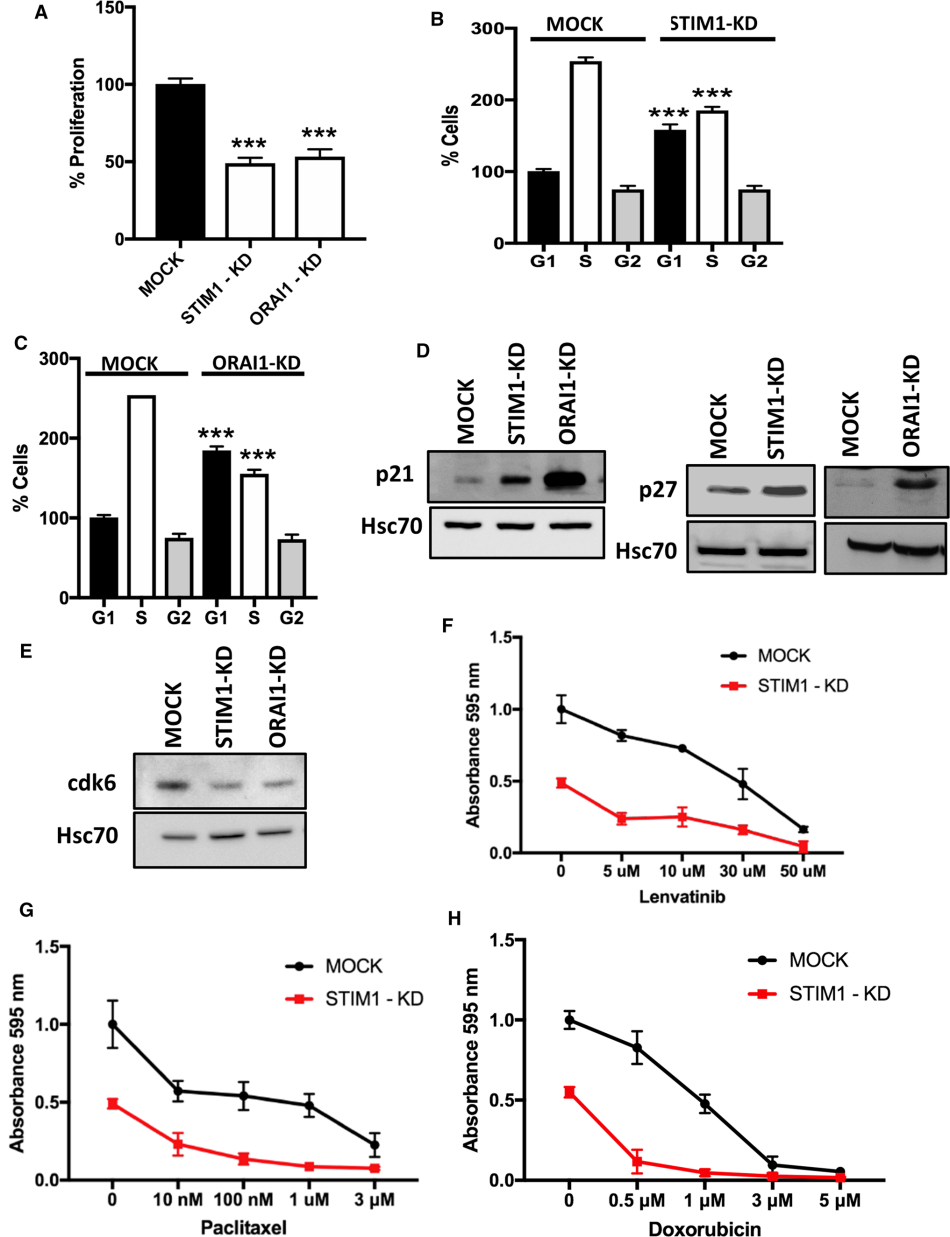
STIM1 and ORAI1 regulate proliferation of thyroid cancer ML-1 cells. **A** STIM1 and ORAI1 knock-down decreased proliferation after 48 h. The bar diagram shows the mean ± S.E. (*n* = 3). ***p < 0.001. **B**, **C** STIM1 and ORAI1 knock-down decreased proliferation by prolonging the G_1_ phase and decreasing the S phases of the cell cycle. ***p < 0.001. *Statistically significant differences between the G_1_ and S phases of cell cycle, respectively, in STIM1-KD and ORAI1-KD cells compared with the MOCK cells. D, Representative Western blots showing the upregulation of p21 and p27 in both STIM1 and ORAI1-KD cells. **E** Representative Western blot showing the downregulation of cyclin-dependent kinase 6 (cdk6) in both STIM1 and ORAI1-KD cells. **F**–**H** Cells were treated with indicated concentrations of Lenvatinib, Paclitaxel or Doxorubicin and the proliferation was assessed by MTT proliferation assays (24 h. The effects of tested drugs were significant (*p* < 0.001) in STIM1-KD compared with MOCK cells. Each data point represents the mean ± S.E. of at least three independent experiments

Evidence have shown that STIM1 and ORAI1 regulate proliferation in different cancers, including brain, ovarian, pancreatic, nasopharyngeal, and renal cancers [32]. We next investigated the effect of STIM1 and ORAI1 knock-down on ML-1 cell proliferation. The results show that the proliferation was significantly attenuated in both STIM1 and ORAI1 knock-down cells, compared with MOCK cells (Fig. 4A). The re-expression of STIM1 in STIM1-KD cells and ORAI1 in ORAI1-KD cells significantly restored the proliferation, compared to MOCK cells (Supplementary Fig. 1C). We next investigated the mechanism by which STIM1 or ORAI1 knock-down cells decreased proliferation. We first analyzed the cell cycle by FACS analysis. In both STIM1-KD and ORAI1-KD cells, there was a significant increase in cell population in the G_1_ phase, and a significant decrease in the S phase of the cell cycle, compared with MOCK cells (Fig. 4B, C). We also investigated the expression of the cell cycle regulatory proteins p21^kip1^and p27^waf1/cip1^, and observed that these proteins were significantly increased in the knock-down cells (Fig. 4D, E). Furthermore, cdk6 was significantly decreased in both STIM1-KD and ORAI1-KD cells, compared with MOCK cells (Fig. 4E).

As we observed a decrease in proliferation in STIM1-KD cells, we further examined if the effect of the chemotherapeutic drugs Lenvatinib, paclitaxel and doxorubicin on the proliferation of MOCK and STIM1-KD ML-1 cells. These drugs attenuated proliferation in a concentration-dependent manner, and the effect was enhanced in STIM1-KD cells (Fig. 4F–H). This is an important observation that suggests a novel approach to design the treatment of thyroid cancer with these drugs.

### STIM1 knock-down affects the migratory signaling pathway, EMT markers and thyroid specific proteins

**Fig. 5:**
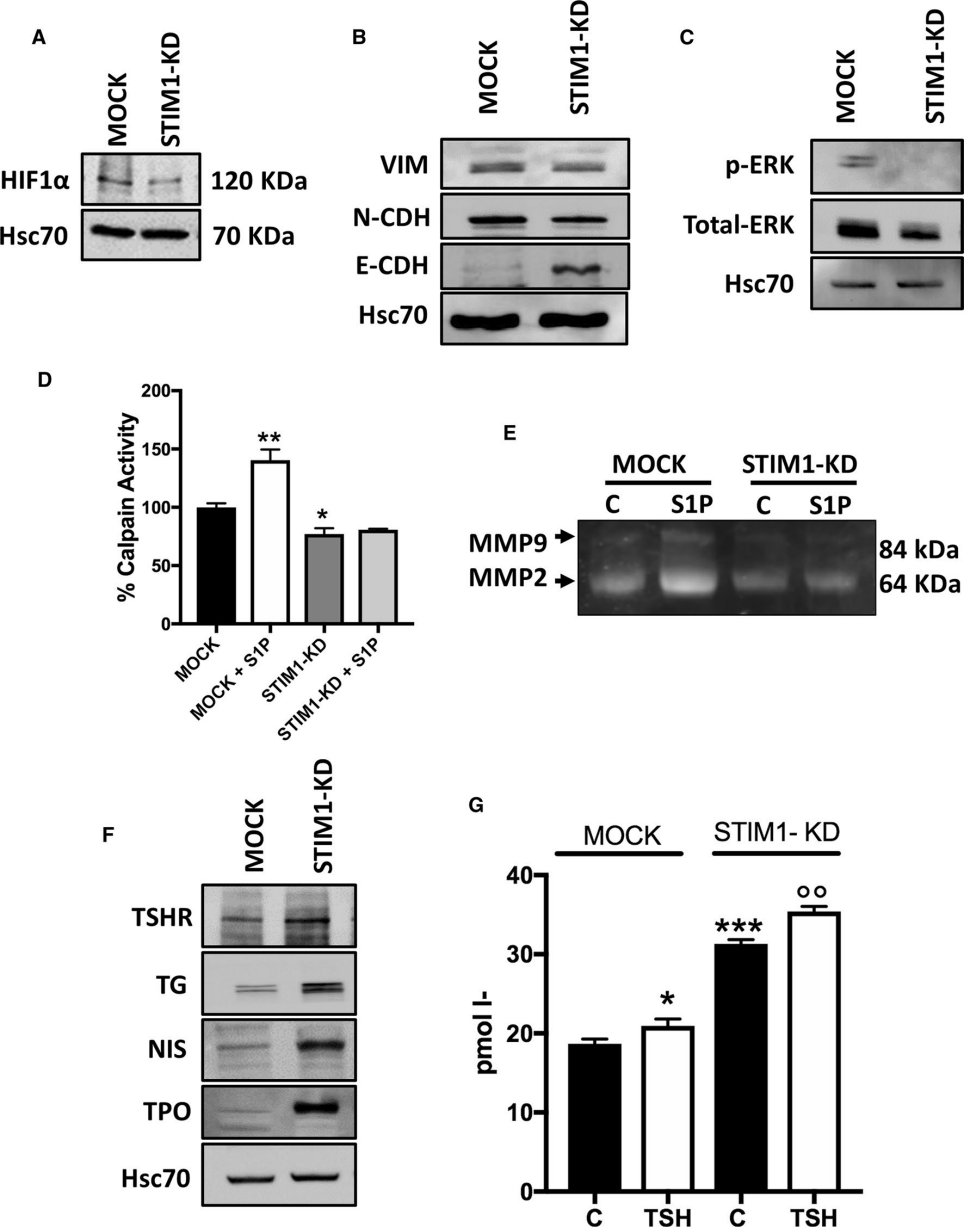
STIM1 knock-down decreased the expression of HIF-1α, calpain activity and the activity of MMP2 and MMP9 and in ML-1 cells. **A** The expression of HIF-1α was significantly decreased in STIM1-KD cells compared with MOCK cells. A representative Western blot is shown. **B** the expression of EMT marker proteins in STIM1-KD cells compared to the MOCK ML-1 cells. The representative Western blots are shown. **C** the expression of total-ERK and phospho-ERK in STIM1-KD cells compared with MOCK ML-1 cells. The representative Western blots are shown. **D** STIM1 knock-down decreased the basal and abolished the S1P-evoked increase in calpain activity compared to MOCK ML-1 cells. (*N* = 4), *Denotes comparison with MOCK. *p < 0.05, **p < 0.01. **E** The basal and S1P evoked gelatinolytic activity of MMP2 and MMP9 was decreased in STIM1-KD cells, compared with MOCK ML-1 cells. The representative zymography blot is shown. **F** STIM1 knock-down increased the expression of thyroid-specific proteins. The representative Western blots of at least three independent experiments are shown. **G** STIM1 knock-down enhanced the basal and TSH-evoked iodine-uptake compared to in the MOCK cells. *Denotes comparison with MOCK cells; o, denotes comparison with control STIM1-KD cells. (*n* = 4) *p < 0.05, ***p < 0.001, ^oo^*p* < 0.01

Previously, we have shown that HIF1α is downregulated in TRPC1 knock-down cells [14]. As can be seen in Fig. 5A, HIF1α expression was significantly downregulated in STIM1-KD cells. Next, we investigated the effects of STIM1-KD on epithelial mesenchymal transition (EMT) marker proteins. We found an upregulation of E-Cadherin protein and a decrease in vimentin and N-Cadherin in STIM1-KD cells, compared to the MOCK ML-1 cells (Fig. 5B). In addition, the expression of total ERK1/2 and phospho-ERK1/2 were downregulated (Fig. 5C). Recently, we have reported that S1P increased calpain activity and the activity of MMP2 and MMP9 through S1P_1,3_, and that knock-down of TRPC1 decreased the secretion and activity of both MMP2 and MMP9 in ML-1 cells [14, 33]. In STIM1-KD cells, the basal calpain activity, and the activity of MMP2 and MMP9 were significantly decreased. Furthermore, the S1P-evoked increase in both calpain activity and the activity of MMP2 and MMP9 was abolished (Fig. 5D, E). This, in part, explains the decreased invasion of STIM1-KD cells.

Thyroid-specific genes, including thyroid-stimulating hormone receptor (TSHR), sodium/iodide symporter (NIS), thyroperoxidase (TPO) and thyroglobulin (TG), are downregulated in thyroid cancer [34]. Therefore, we examined the expression of these proteins in STIM1-KD cells. Interestingly, these proteins were upregulated in STIM1-KD cells (Fig. 5F). In addition, both the basal and TSH-evoked (final concentration 1 mU/ml) iodine-uptake was increased in STIM1-KD cells (Fig. 5G).

### MPDA-DMEA nanoparticles, an efficient siRNA delivery system in ML-1 cells

**Fig. 6.**
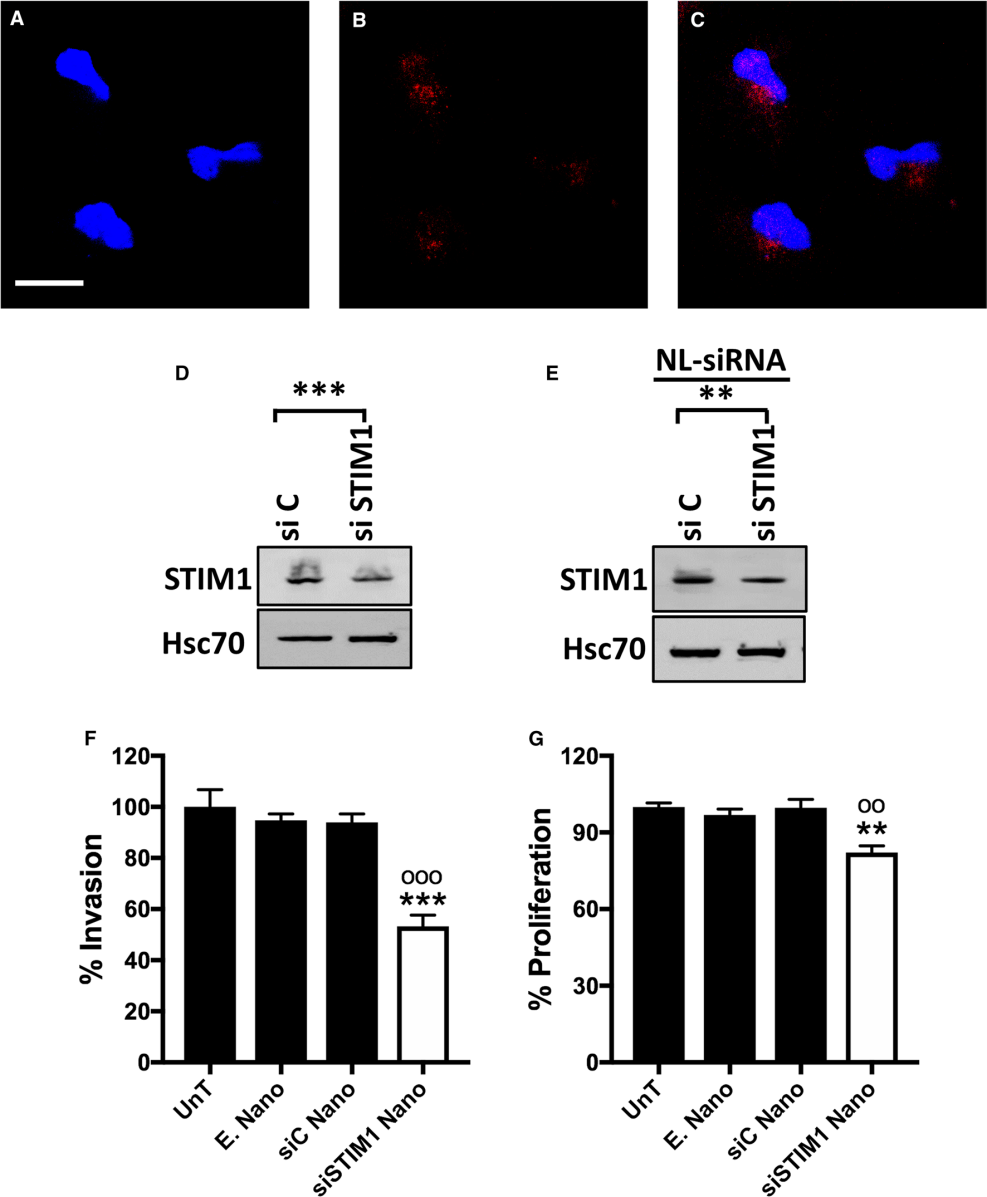
Functional efficacy of MPDA-DMEA nanoparticles in ML-1 thyroid cancer cells. **A**–**C** Confocal images of ML-1 cells with control siRNA-Alexa Fluor 555 loaded nanoparticles. **A** (DAPI), **B** (siRNA-Alexa Fluor 555), and **C** (Merge: DAPI + siRNA-Alexa Fluor 555). The scale bar is 20 μm. The images shown are representative of at least three separate experiments. **D** Representative western blot showing the efficacy of siSTIM1 in ML-1 cells. **E** Representative western blot showing the efficacy of siSTIM1-loaded MPDA-DMEA nanoparticles in ML-1 cells. NL-siRNA denotes nanoparticles loaded siRNA. **F**, **G** siSTIM1-loaded nanoparticles decreased the invasion and proliferation of ML-1 cells. UnT, un-treated; E.Nano, empty MPDA-DMEA nanoparticles, siC Nano, control non-target siRNA loaded nanoparticles, siSTIM1 Nano, siSTIM1 loaded nanoparticles. *ML-1 unT vs siSTIM1 Nano, ^o^siC Nano vs siSTIM1 Nano. The *bars *show the mean ± S.E. (*n* = 6). **p < 0.01; ***p < 0.001; ^oo^*p* < 0.01; ^ooo^*p* < 0.001

Nanoparticles have been shown to deliver siRNA or drugs into thyroid cancer cells precisely and effectively [35, 36]. Therefore, we first tested the uptake and release of control siRNA Alexa 555 Fluor-loaded MPDA-DMEA nanoparticles in ML-1 thyroid cancer cells. These nanoparticles were efficiently taken up by the cells and the siRNA was released after 96 h (Fig. 6A-C). In other control experiments, we showed that transfecting with STIM1-siRNA, downregulate STIM1 protein in ML-1 cells (Fig. 6D). We observed the same effect using STIM1-siRNA loaded MPDA-DMEA nanoparticles (Fig. 6E). Next, we investigated the effect of the empty, or STIM1-siRNA loaded nanoparticles on ML-1 cell invasion and proliferation. There was no effect of empty nanoparticles, however, both the invasion and proliferation were significantly downregulated by STIM1-siRNA loaded nanoparticles (Fig. 6F, G).

### Zebrafish embryos with human thyroid tumor cells-xenograft

**Fig 7:**
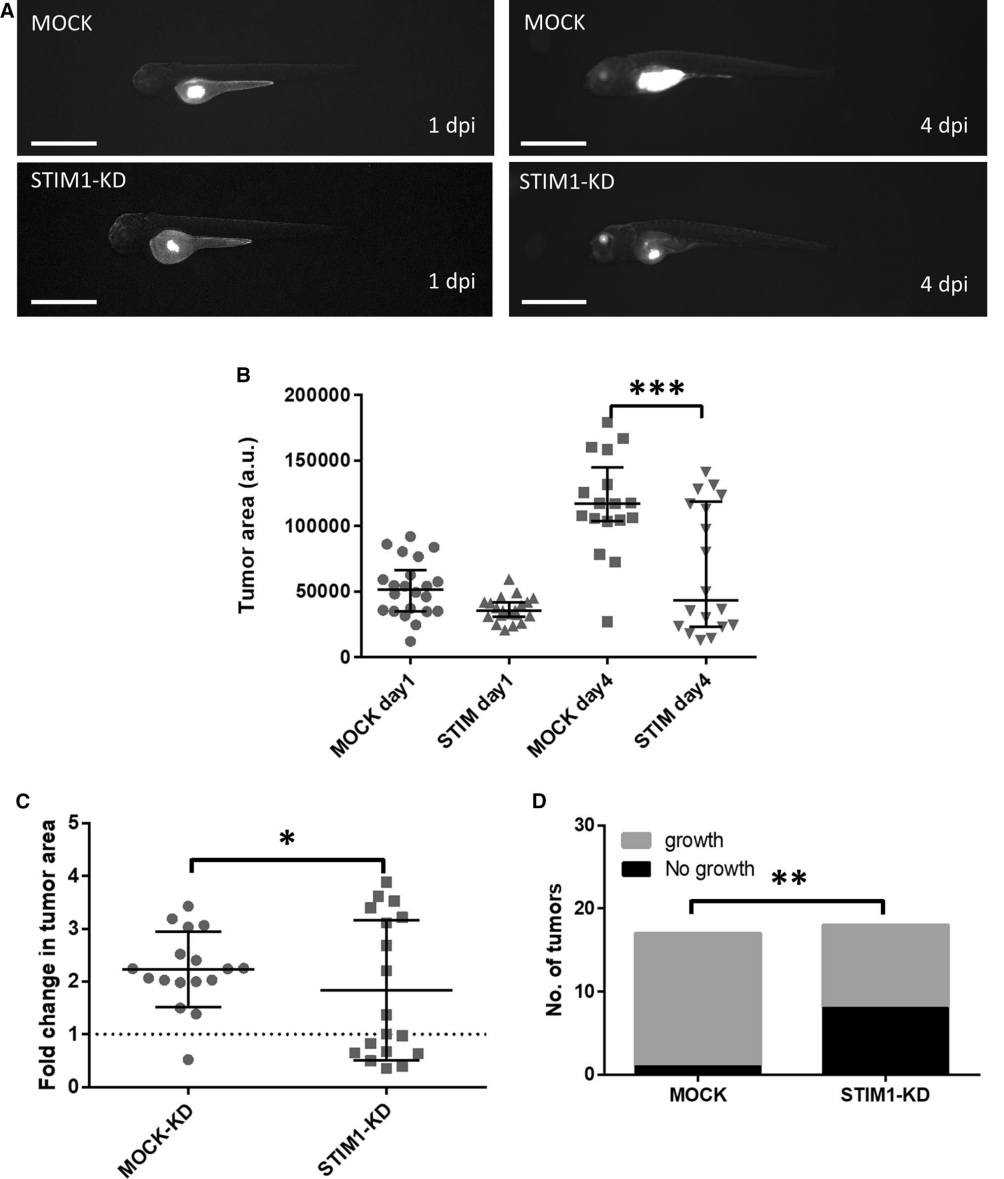
Zebrafish xenograft experiments. **A** Representative images of one and four day (s) post injection (dpi) with MOCK and STIM1-KD cells and the increase in tumor growth. The scale bar is 1 mm. **B**, **C** Quantitation of tumor size and fold change tumor area. **D** Quantitation of tumor growth. *p < 0.05; **p < 0.01; ***p < 0.001

To analyze the effects of STIM1 knock-down on tumor growth in vivo, we transplanted MOCK and STIM1-KD ML-1 cells into the zebrafish embryos. The embryos were kept in 96-well plates (1 embryo/well) to allow longitudinal analysis of tumor growth. STIM1-KD ML-1 cells exhibited strongly reduced tumor growth, also confirmed by calculating the tumor area and fold change (Fig. 7A–D). Taken together, these data indicate STIM-1 is required for full tumorigenic potential of ML-1-cells. Furthermore, to discriminate between a halt in the proliferation versus increased apoptosis upon STIM1 knockdown, we used anti-activated Caspase 3 antibody immunofluorescence staining of MOCK and STIM1-KD xenografts. The results showed that the Caspase 3 signal was significantly higher in STIM1-KD xenograft cells compared to the MOCK xenograft cells (Supplementary Fig. 2. A, B). These findings conclude that the STIM1 knockdown significantly induce apoptosis in ML-1 xenograft cells.

In addition, to confirm that the quantified CellTracker Green signal in zebrafish xenografts is coming from living human thyroid tumor cells, we performed human specific Ki67 antibody immunofluorescence staining. The results showed that the CellTracker Green signal was originating from human thyroid tumor living cells. The quantification of Ki67 antibody signal in MOCK xenograft tumor was significantly higher compared to in STIM1-KD xenograft tumor (Supplementary Fig. 2. C, D). These findings conclude that the MOCK xenograft cells are dividing actively while STM1-KD xenograft cells are not proliferating efficiently which in part is due to induction of apoptosis.

### Analysis of STIM1 and ORAI1 gene and protein expression in human thyroid

#### Expression of STIM1 in normal and thyroid cancer patient samples

**Fig. 8:**
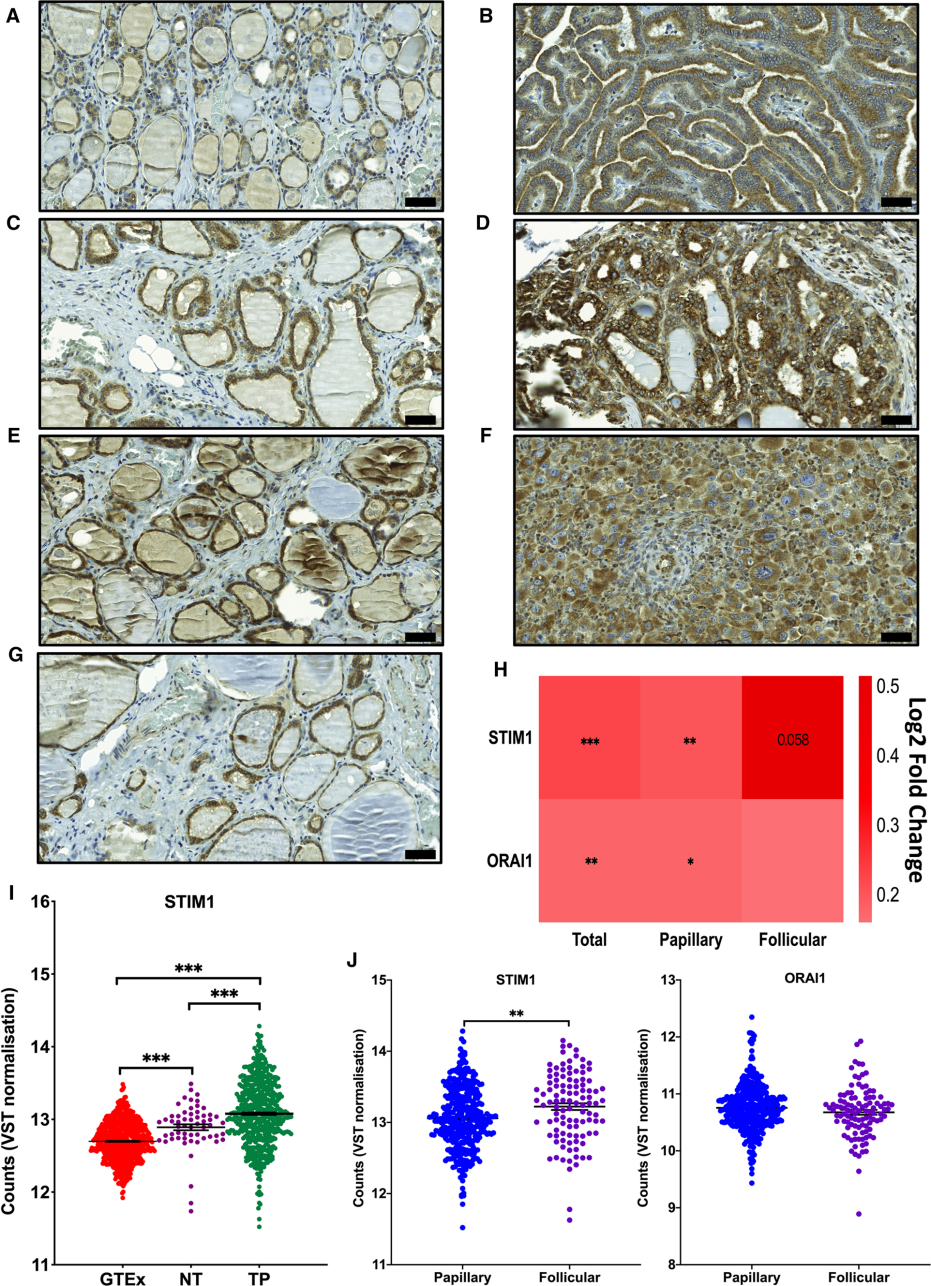
STIM1-protein expression in human normal and thyroid cancer tissues. **A**, **B** Representative images of normal and papillary thyroid cancer. **C**, **D** Representative images of normal and follicular thyroid cancer tissue. **E**, **F** Representative images of normal and anaplastic thyroid cancer tissue. The representative images of control and cancer tissue sections of each cancer type are from the same patient. **G** Representative image of normal thyroid tissue from a healthy subject. Scale bar 50 µM. The final images were acquired at 20× magnification using case viewer software (3DHITECH Ltd, Germany). **H** Heatmap to visualize differential expression of STIM1 and ORAI1 between thyroid tumors and their adjacent normal samples. The color intensity represents log2 Fold Changes. Significances have been corrected by Benjamini–Hochberg method. *p < 0.05; **p < 0.01; ***p < 0 .001. **I** STIM1 expression comparison between thyroid cancer tissues, their para-cancerous tissues, and normal controls. Data were presented as mean ± SE. One-way ANOVA test was used to assess statistical significance. ***p < 0 001. **J** STIM1 and ORAI1 expression comparison between papillary thyroid cancer tissues and follicular thyroid cancer tissues. Data were presented as mean ± SE. Unpaired *t* test was used to assess statistical significance. **p < 0.01.

A higher expression of STIM1 has been suggested to correlate with poor prognosis and survival in many cancers [37]. We investigated the expression of STIM1 in thyroid cancer patient samples and obtained 176 thyroid cancer patient tissue samples and 104 normal thyroid tissue samples (normal tissue of the same thyroid cancer patient, as well as normal thyroid tissue from healthy subjects) as described in the method section. The results showed an increased STIM1 staining in all types of thyroid cancer patient samples, compared to respective patient’s normal thyroid tissue adjacent to the tumor, or with healthy subjects’ normal thyroid tissue sections (Fig. 8A–G).

### Differential gene expression in thyroid cancer versus normal thyroid tissues

STIM1 and ORAI1 gene expression was evaluated in thyroid tumor tissues (*n* = 502) versus normal thyroid solid tissues (*n* = 58) datasets in the TCGA Thyroid Carcinoma cohort. The expression of both STIM1 and ORAI1 genes was significantly upregulated in both the papillary and combined thyroid cancer tissues (Total), compared to tumor adjacent normal thyroid tissues (NT). However, we found STIM1 borderline upregulated (*p* = 0.058) but not ORAI1 in follicular thyroid cancer tissues (Fig. 8H). Furthermore, a comparison of STIM1 expression in normal thyroid solid tissues (GTEX dataset), thyroid tumor tissues (TP) and tumor adjacent normal thyroid tissues (NT) was determined. Interestingly, we observed that STIM1 was significantly upregulated not only in thyroid tumor tissues (TP) but also in the tumor adjacent thyroid tissues (NT), compared to the normal thyroid tissues (GTEX) (Fig. 8I). In another comparison, we found the STIM1 expression was significantly higher in follicular thyroid cancer tissues compared to the papillary thyroid cancer tissues (Fig. 8J).

## Discussion

The hallmarks of cancer include uncontrolled growth, and invasion of the cancer cells [38, 39]. For these processes calcium and calcium channels have been shown to play important roles [11, 40, 41]. We have previously shown that calcium entry is important for the invasion and proliferation of follicular thyroid cancer ML-1 cells [14]. The STIM- and ORAI proteins have also been considered as novel targets for cancer therapy [15, 32]. We show here that, especially STIM1, but to some extent also ORAI1, are upregulated in several thyroid cancers cell lines, compared with normal thyroid cells. This has also been shown in e.g. therapy resistant ovarian cancer cell lines [42]. Furthermore, STIM1 was upregulated in all studied patient samples of papillary, follicular, and anaplastic thyroid cancer, compared with healthy thyroid tissues from the same patients or from normal healthy subjects. Similar results have been obtained in several other cancers, including cervical cancer [43], melanoma [44], and colorectal cancer [45]. The differential STIM1 and ORAI1 gene expression analysis data clearly showed an upregulation of these genes in most of the thyroid cancer tissues compared to normal thyroid tissues datasets. Surprisingly, an increased STIM1 gene expression but not ORAI1 was observed in the tumor adjacent normal tissue, compared to the normal thyroid tissue, indicating a possible role of STIM1 in the initiation of thyroid tumor formation. A higher STIM1 expression in follicular thyroid cancer tissues compared to the papillary thyroid cancer tissues datasets further suggests that STIM1 is important in the development of an aggressive thyroid cancer phenotype.

Downregulation of STIM1 or ORAI1 efficiently attenuated both proliferation and invasion of ML-1 cells, decreased the expression of cdk6 and increased the expression of the cell cycle regulators p21 and p27. In cervical cancer, inhibition of STIM1 resulted in increased expression of p21, and arrested the cells in the S- and G2/M phase of the cell cycle [43]. In our experiments STIM1-KD and ORAI1-KD halted cells in the G1 and S phase. This was also observed in TRPC1-KD ML-1 cells [14]. We next investigated the effects of STIM1-KD in Zebrafish embryos [46, 47]. By injecting control ML-1 cells in the zebrafish xenograft experiments, the cells grew into a clear tumor; however, the STIM1-KD cells were unable to evoke tumor growth. This experiment shows that also in an in vivo setting, STIM1-KD cells were unable to proliferate and form a tumor.

In addition to basal invasion, STIM1 and ORAI1 knock down cells reduced the S1P-evoked invasion. As shown previously, S1P is an important enhancer of cancer cell invasion [48], including thyroid cancer cells [16]. In both knock down cell lines, the expression of VEGFR2 was decreased, whereas SIP3 was decreased in STIM1-KD cells, and S1P1 in ORAI1-KD cells. These receptors are crucial for S1P-evoked invasion [49]. In melanoma, STIM1 blockade the recycling of MT1-MMP to the plasma membrane, thus inhibiting the breakdown of the extracellular matrix [44]. Furthermore, in STIM1-KD cells the S1P-stimulated extrusion of MMP2 and -9 was attenuated, and HIF-1α, an important regulator of ML-1 cell invasion [50], was reduced. The results are in line with our previous study, showing that in TRPC1-KD ML-1 cells, the expression of VEGFR2, S1P3, MMP2 and -9, and HIF-1a were all decreased [14]. Collectively these results show that calcium fluxes play a vital role for the regulation of factors involved in the proliferation and invasion of thyroid cancer cells.

In addition to surgery, pharmacological interventions are important in treating thyroid cancer [51]. We show that three commonly used drug, i.e., lenvatinib, paclitaxel and doxorubicin, efficiently attenuated proliferation of the ML-1 cells. Notably, the effect of these drugs was enhanced in STIM1-KD cells. A similar observation was made in STIM1-KD pancreatic adenocarcinoma cells [52]. These results suggest that a combination of chemotherapy and knock down or blocking of STIM could be effective in treating thyroid cancer. However, the use of pharmacological blockers or siRNA in the inhibition of e.g., STIM1 is problematic, considering the ubiquitous expression of this protein. To avoid this, nanoparticles carrying drugs or siRNA can be an option [53]. This has been proposed for thyroid cancer cells [35, 54]. We have previously shown that drug-loaded nanoparticles can efficiently induce apoptosis of thyroid cancer cells [36]. Here we show that siRNA-loaded nanoparticles can be used successfully to knock down STIM1, and thereby attenuate both proliferation and invasion of the ML-1 cells. A combination of siRNA-loaded nanoparticles and lower doses of e.g., doxorubicin could be a novel approach to curtail thyroid cancer progression, with less adverse effects of the chemotherapy.

An interesting observation was the fact that knocking down STIM1 increased the TSH receptor expression. In rat thyroid FRTL-5 cells, knock down of TRPC2 also induced an increase in TSH receptor expression [17]. Further, we observed an increased expression of the thyroid specific proteins thyroglobulin, thyroperoxidase, and the Na-I symporter in STIM1-KD cells. STIM1 knock down enhanced TSH-evoked uptake of iodine, decreased vimentin and N-cadherin expression, and elevated E-cadherin expression. Previously, knock-down of STIM1 has been shown to suppress the expression of proteins important for epithelial-mesenchymal transition and enhance the expression of E-cadherin in human prostate cancer cells [55]. Our results thus suggest that the ML-1 cells obtained a more normal thyroid phenotype, with re-differentiation of the cells [56]. The precise mechanisms by which STIM1-KD regulates the expression of these proteins remain to be established. Notably, as STIM1-KD increased TSH-evoked uptake of iodine, the cells should be more susceptible to take up radioactive iodine, which may induce apoptosis. Thus, radioactive iodine could be used to destroy thyroid cancer cells.

## Conclusions

We have shown here using different approaches and gene downregulation in thyroid cancer cells that calcium signaling and the STIM1 protein play an important function in driving thyroid cancer cell proliferation and invasion. Furthermore, we have shown that blocking calcium entry pathways by using siRNA-loaded nanoparticles effectively inhibit proliferation and invasion of the cells. In view of this, we propose that the combination of drugs and siRNA targeting could be a useful approach to design better treatments for thyroid cancer and for the benefit of patients afflicted by aggressive, metastatic forms of thyroid cancer.

### Supplementary Information

Below is the link to the electronic supplementary material.

Supplementary Figure 1. Re-Expression Of Stim1 Or Orai1 In Stim1-Kd And Orai1-Kd Cells Rescues Both Invasion And Proliferation. A, Expressing Stim1 (Pstim1) Back To Stim1-Kd Cells Rescues Both Basal And S1p-Evoked (Final Concentration, 100 Nm) Invasion. *, Statistically Significant Differences In Migration Compared With Mock Cells; O, Statistically Significant Differences In Migration Of Stim1-Kd Compared With Stim1-Kd + Pstim1; Ö, Statistically Significant Differences In Migration Of Stim1-Kd + Pstim1 Compared With Stim1-Kd + Pstimi1 + S1p. The Normalized Results In The Graphs Are The Means ± S.E. (N = 3). ***, *p* < 0.001; Oo, *p* < 0.01; Öö, *p* < 0.01. B, Expressing Orai1 (Porai) Back To Orai1-Kd Cells Rescues Both Basal And S1p-Evoked (Final Concentration, 100 Nm) Invasion. *, Statistically Significant Differences In Migration Compared With Mock Cells; O, Statistically Significant Differences In Migration Of Orai1-Kd Compared With Orai1-Kd + Porai1; Ö, Statistically Significant Differences In Migration Of Orai1-Kd + Porai1 Compared With Orai1-Kd + Porai1 + S1p. The Normalized Results In The Graphs Are The Means ± S.E. (n = 3). ***, *p* < 0.001; *, *p* < 0.05; Oo, *p* < 0.01; Ö, *p* < 0.05. C, Expression Of Stim1 Or Orai1 In Stim1-Kd Or Orai1-Kd Cells Respectively, Restored Proliferation After 24 H. The Bar Diagram Shows The Means ± S.E. *, The Statistically Significant Differences In Proliferation Compared With Mock Cells; O, The Statistically Significant Differences In Proliferation Of Stim1-Kd Cells Compared With Stim1-Kd + Pstim1 Cells; Ö, The Statistically Significant Differences In Proliferation Of Orai1-Kd Cells Compared With Orai1-Kd + Porai1 Cells. The Normalized Results In The Graph Are The Means ± S.E. (*N* = 3). ***, *p* < 0.001; *, *p* < 0.05; Ooo, *p* < 0.001; Ööö, *P* < 0.001.Upplementary File1 (Tif 883 Kb)

Supplementary Figure 2. Validation Of Celltracker Green Signal And Analysis Of Apoptosis In Thyroid Cancer Ml-1 Cells Xenografts. Xenografted Fixed Embryos Were Immuno-Stained As Whole-Mounts For Celltracker Green And Cleaved Caspase 3 And Imaged With A Confocal Microscope. A) Confocal Images Of Tumours Immunostained With Anti-Cleaved Caspase 3. Tumor Area Was Outlined With A White Dashed Line. Scale Bar 100um. B) The Average Cleaved Caspase Signal Of The Tumor Was Measured And Data Analysed Statistically With Nonparametric Mann-Whitney Test. Number Of Samples: No Tumor, n = 6 ; Mock-Kd, n=7 ; Stim1-Kd n=6. Median And Interquartile Range Together With Individual Datapoints Is Plotted. C) Confocal Images Of Tumours Immunostained With Anti-Ki67 Antibody. Tumor Area Was Outlined With A White Dashed Line. Scale Bar 100um. D) The Average Ki67 Signal Of The Tumor Was Measured And Data Analysed Statistically With Nonparametric Mann-Whitney Test. Number Of Samples: No Tumor, n=5 ; Mock-Kd, n=5 ; Stim1-Kd n=6. Median And Interquartile Range Together With Individual Datapoints Is Plotted. *p* Values < 0.05 Were Considered Statistically Significant.File2 (Tif 5276 Kb)

Supplementary file3 (TIF 653 KB)

## Data Availability

All data analyzed during this study are included in this published article [and its supplementary information files].
