# Supplementary figures and images for: Stromal interaction molecule 1 (STIM1) knock down attenuates invasion and proliferation and enhances the expression of thyroid-specific proteins in human follicular thyroid cancer cells
Source: Cell Mol Life Sci. 2021 Jun 21;78(15):5827–46. doi: 10.1007/s00018-021-03880-0 (PMC8316191; doi:10.1007/s00018-021-03880-0)

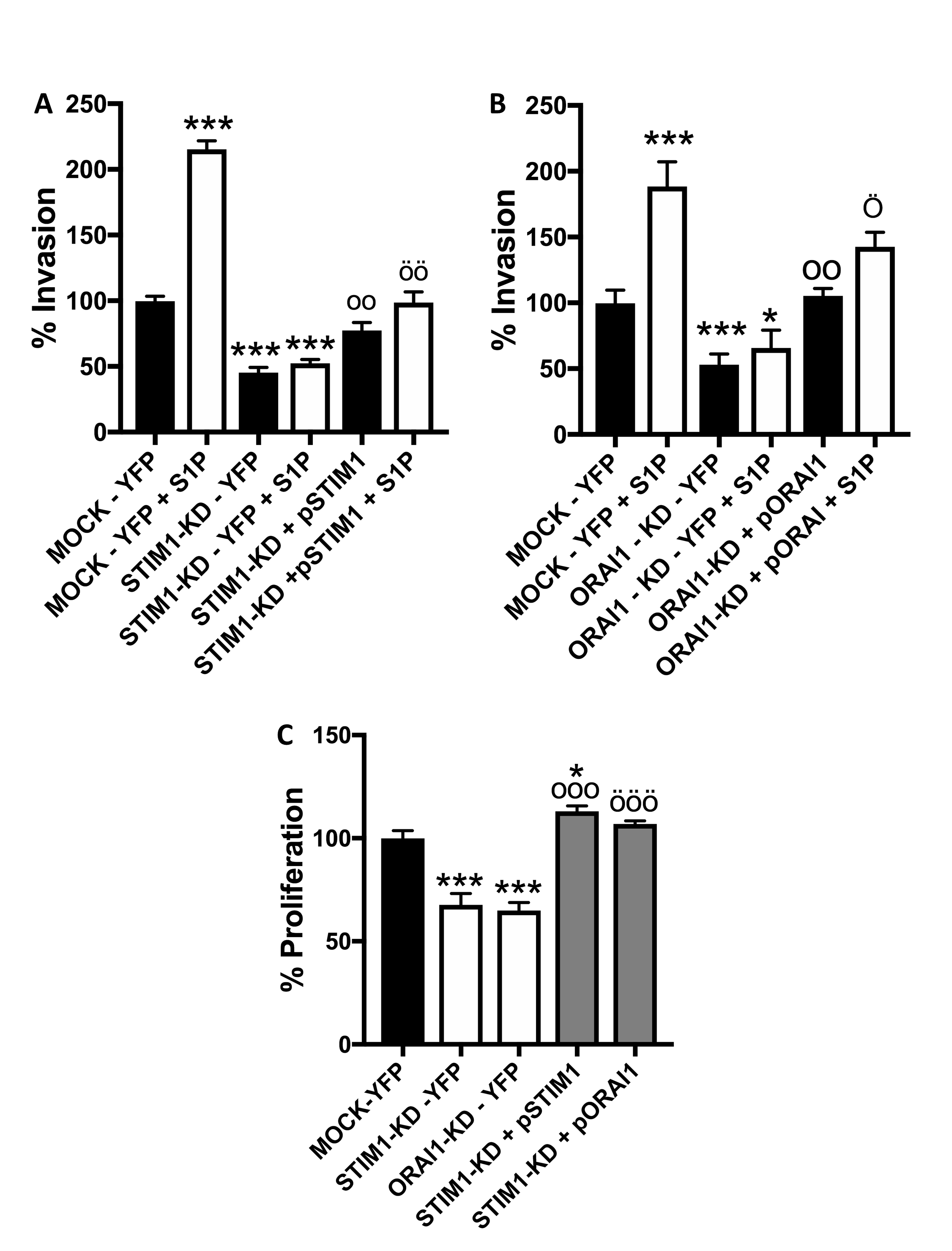

Supplement: Supplementary file 1 — Supplementary Figure 1. Re-Expression Of Stim1 Or Orai1 In Stim1-Kd And Orai1-Kd Cells Rescues Both Invasion And Proliferation. A, Expressing Stim1 (Pstim1) Back To Stim1-Kd Cells Rescues Both Basal And S1p-Evoked (Final Concentration, 100 Nm) Invasion. *, Statistically Significant Differences In Migration Compared With Mock Cells; O, Statistically Significant Differences In Migration Of Stim1-Kd Compared With Stim1-Kd + Pstim1; Ö, Statistically Significant Differences In Migration Of Stim1-Kd + Pstim1 Compared With Stim1-Kd + Pstimi1 + S1p. The Normalized Results In The Graphs Are The Means ± S.E. (N = 3). ***, p < 0.001; Oo, p < 0.01; Öö, p < 0.01. B, Expressing Orai1 (Porai) Back To Orai1-Kd Cells Rescues Both Basal And S1p-Evoked (Final Concentration, 100 Nm) Invasion. *, Statistically Significant Differences In Migration Compared With Mock Cells; O, Statistically Significant Differences In Migration Of Orai1-Kd Compared With Orai1-Kd + Porai1; Ö, Statistically Significant Differences In Migration Of Orai1-Kd + Porai1 Compared With Orai1-Kd + Porai1 + S1p. The Normalized Results In The Graphs Are The Means ± S.E. (n = 3). ***, p < 0.001; *, p < 0.05; Oo, p < 0.01; Ö, p < 0.05. C, Expression Of Stim1 Or Orai1 In Stim1-Kd Or Orai1-Kd Cells Respectively, Restored Proliferation After 24 H. The Bar Diagram Shows The Means ± S.E. *, The Statistically Significant Differences In Proliferation Compared With Mock Cells; O, The Statistically Significant Differences In Proliferation Of Stim1-Kd Cells Compared With Stim1-Kd + Pstim1 Cells; Ö, The Statistically Significant Differences In Proliferation Of Orai1-Kd Cells Compared With Orai1-Kd + Porai1 Cells. The Normalized Results In The Graph Are The Means ± S.E. (N = 3). ***, p < 0.001; *, p < 0.05; Ooo, p < 0.001; Ööö, P < 0.001.Upplementary File1 (Tif 883 Kb) [file 18_2021_3880_MOESM1_ESM.tif]

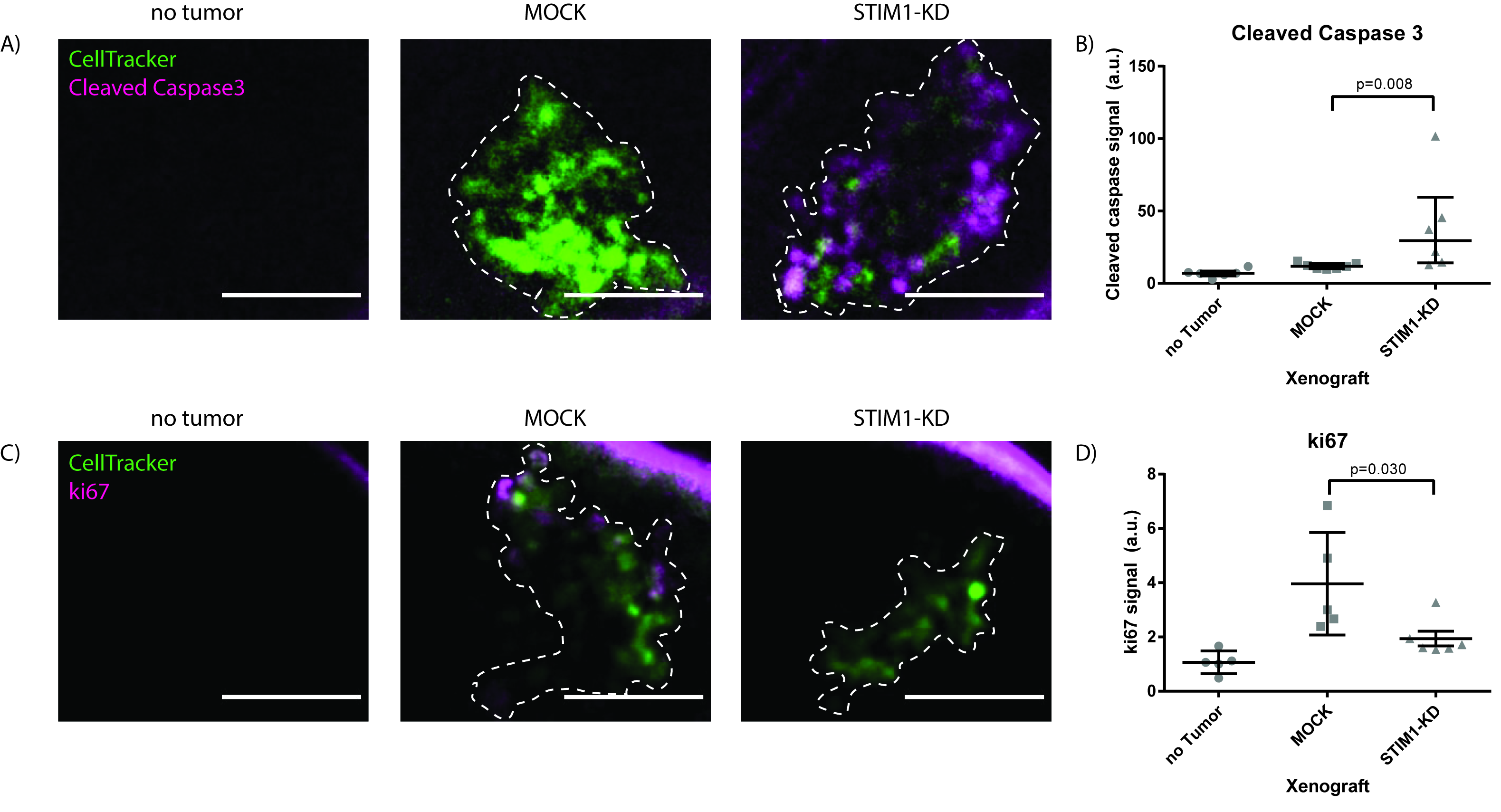

Supplement: Supplementary file 2 — Supplementary Figure 2. Validation Of Celltracker Green Signal And Analysis Of Apoptosis In Thyroid Cancer Ml-1 Cells Xenografts. Xenografted Fixed Embryos Were Immuno-Stained As Whole-Mounts For Celltracker Green And Cleaved Caspase 3 And Imaged With A Confocal Microscope. A) Confocal Images Of Tumours Immunostained With Anti-Cleaved Caspase 3. Tumor Area Was Outlined With A White Dashed Line. Scale Bar 100um. B) The Average Cleaved Caspase Signal Of The Tumor Was Measured And Data Analysed Statistically With Nonparametric Mann-Whitney Test. Number Of Samples: No Tumor, n = 6 ; Mock-Kd, n=7 ; Stim1-Kd n=6. Median And Interquartile Range Together With Individual Datapoints Is Plotted. C) Confocal Images Of Tumours Immunostained With Anti-Ki67 Antibody. Tumor Area Was Outlined With A White Dashed Line. Scale Bar 100um. D) The Average Ki67 Signal Of The Tumor Was Measured And Data Analysed Statistically With Nonparametric Mann-Whitney Test. Number Of Samples: No Tumor, n=5 ; Mock-Kd, n=5 ; Stim1-Kd n=6. Median And Interquartile Range Together With Individual Datapoints Is Plotted. p Values < 0.05 Were Considered Statistically Significant.File2 (Tif 5276 Kb) [file 18_2021_3880_MOESM2_ESM.tif]

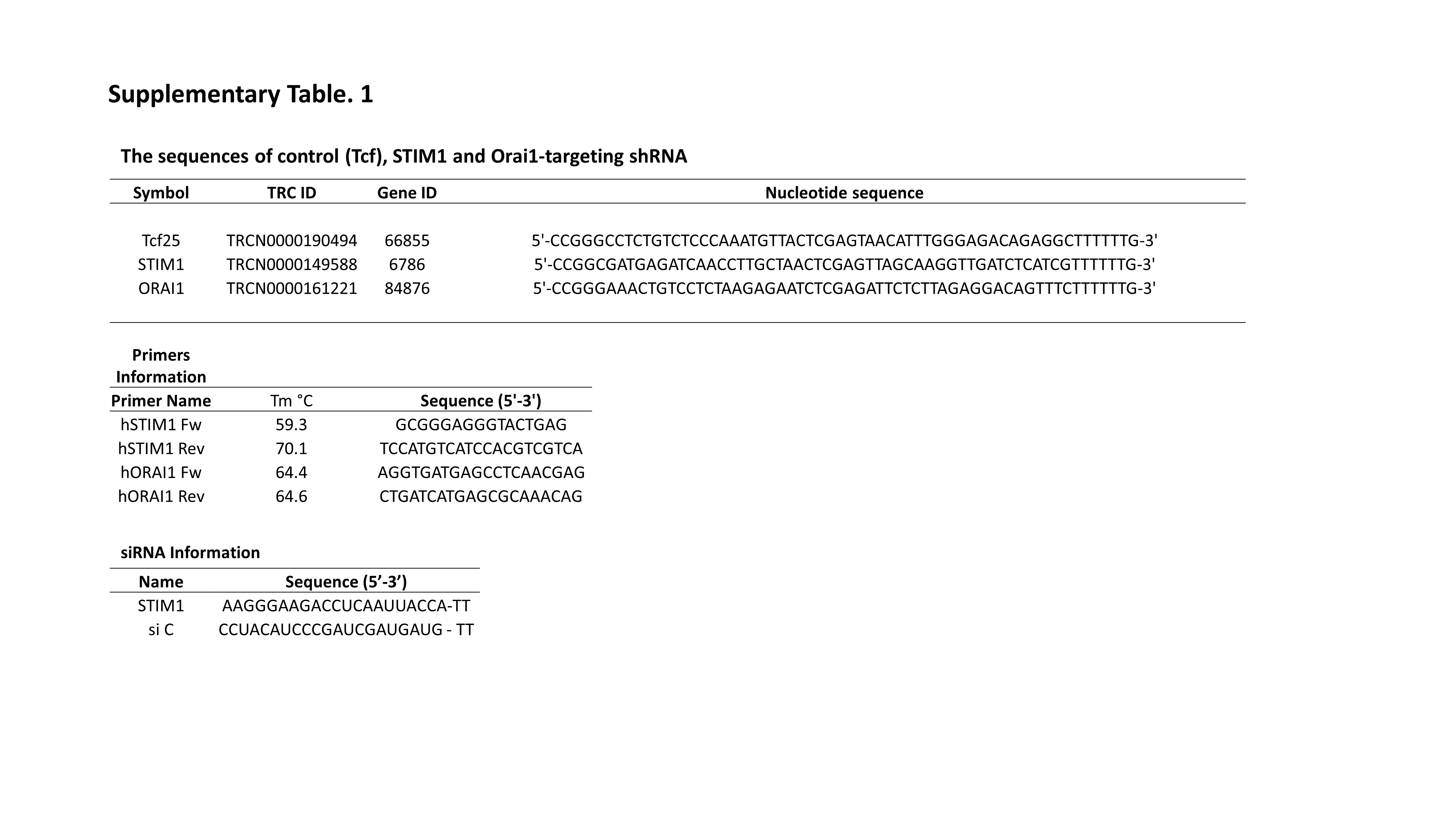

Supplement: Supplementary file 3 — Supplementary file3 (TIF 653 KB) [file 18_2021_3880_MOESM3_ESM.tif]
